# Using phase response curves to predict synchronization times for neural circuits

**DOI:** 10.1186/1471-2202-16-S1-P39

**Published:** 2015-12-18

**Authors:** Patrick Crotty

**Affiliations:** 1 Department of Physics and Astronomy, Colgate University, Hamilton, NY 13346, USA

## 

In a previous work [1], it was found that small simulated circuits of regularly spiking entorhinal cortex layer II stellate cells (using the model of [2]) synchronize fastest when their intrinsic firing frequencies are approximately in the 15-20 Hz range, which is very near the θ frequency range (8-12 Hz) where these cells are experimentally known to actually fire. The synchronization time (which we define as the mean time after the onset of synaptic coupling it takes the cells to synchronize their firings to within one action potential width of each other, starting with an initially random phase configuration) in this optimal frequency range can be several times lower than when the cells have either higher or lower intrinsic frequencies, is robust across a wide range of 2- and 3-cell circuit topologies and synaptic coupling strengths, and appears for both excitatory and inhibitory coupling. The existence of such an optimization may be significant both for the entorhinal cortex itself (where a background θ rhythm is believed to play a role in the phase-coding of position information by grid cells [3]) and in other parts of the brain for which cell assemblies play an essential role in information processing, in that assemblies of intrinsically θ-frequency cells would be able to form much faster than assemblies of other cells.

**Figure 1:**
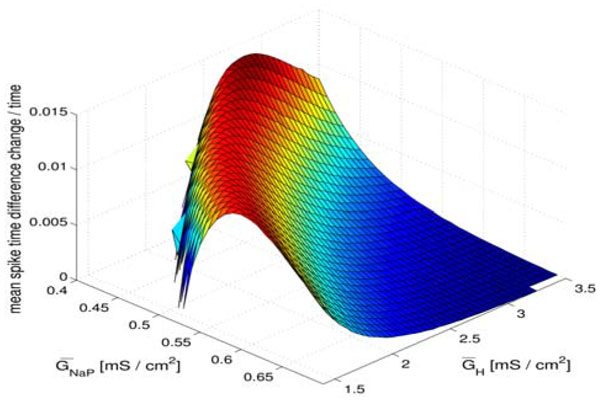
**The mean value of the STDM divided by the period (the combination is dimensionless) as a function of the *h *and persistent sodium conductances in the stellate cell, which are the parameters most influencing the intrinsic spiking frequency**. The region of maximal values (red) is approximately in the θ and low-β region of intrinsic spiking frequency, and also approximately the frequency range where circuits of simulated cells are observed to synchronize most quickly and where real stellate cells lie. The results shown are for excitatory coupling with synaptic conductance 0.01 mS / cm^2^.

The spike-time difference map (STDM) formalism of [2] uses the phase response curves (PRCs) of two identical coupled cells to predict the existence and stability of synchronized and other steady-state firing patterns. The STDM is an iterate of the PRC which gives the amount by which the time between corresponding spikes in the two cells changes from one cycle to the next. By taking the mean value of the STDM and dividing by the period, which gives essentially the mean "rate" at which the spike time difference is changing, we find a prominent band of maxima in the same frequency region as the synchronization time minima (Fig. 1). Like the synchronization time minimization, the existence and location of this region of maxima appears to be relatively insensitive to synaptic coupling strength and excitatory versus inhibitory coupling. Thus, the STDM may provide the basis for a semi-analytical approach for finding the regions of parameter space most favorable for synchronization time.
